# Optimization of Selection Agent Concentrations and Expanding G418 Utility for Gentamicin Resistance in Marchantia polymorpha

**DOI:** 10.21203/rs.3.rs-5333121/v1

**Published:** 2024-11-28

**Authors:** Andisheh Poormassalehgoo, Elżbieta Kaniecka, Mohamadreza Mirzaei, Shino Goto-Yamada

**Affiliations:** Malopolska Centre of Biotechnology, Jagiellonian University

**Keywords:** Marchantia polymorpha, genetic transformation, herbicide, aminoglycoside antibiotics, cross activity

## Abstract

Genetic transformation of plants is pivotal for advancing biotechnology, with success depending largely on effective selection methods. *Marchantia polymorpha* has emerged as a model plant due to its evolutionary importance, ease of manipulation, and simple genetic structure. However, inconsistent antibiotic performance and limited studies on optimal selection agent concentrations have posed challenges. This study aimed to optimize selection agent use in *M. polymorpha* genetic transformation. We assessed the effects of five antibiotics (hygromycin, kanamycin, G418, neomycin, and gentamicin) and the herbicide chlorsulfuron on *M. polymorpha* gemmae growth. For each agent, we identified the minimum lethal concentration for nontransgenic plants and safe thresholds for transgenics, balancing false-positive prevention with reduced toxicity. Hygromycin, G418, and chlorsulfuron exhibited broad selective concentration ranges, enabling efficient transformant selection. Notably, we observed cross-activity of the gentamicin resistance enzyme with G418, a phenomenon also seen in tobacco. This study effectively determined optimal concentrations of selective agents for *M. polymorpha* gemmae transformation. Additionally, the unexpected cross-activity underscores the need for careful marker selection and highlights potential for strategic antibiotic use. Our findings enhance transformation protocols for *M. polymorpha* and possibly other plant species.

## Background

Bryophytes, including the liverwort *Marchantia polymorpha*, are nonvascular plants that diverged from the lineage leading to modern flowering plants over 400 million years ago. These plants are valuable for research due to their role in preventing soil erosion, contributing to soil formation, stabilization, humus accumulation, and water retention. Bryophytes also provide nutritional requirements for various organisms, such as insects, millipedes, and earthworms [1]. Beyond their ecological significance, these plants are useful tools for plant biological research due to their unique features. *M. polymorpha*, characterized by simple morphology, low genetic redundancy, a haploid-dominant life cycle, availability of both sexual and vegetative propagation, and rapid growth, has become an excellent experimental material for plant biological studies, providing insights into plant evolution and diversification [2, 3].

Genetic transformation introduces foreign DNA into an organism’s genome to modify its genes. For *M. polymorpha*, methods such as Agrobacterium-mediated transformation, particle bombardment (biolistics), PEG-mediated protoplast transformation, and electroporation have been proposed [3]. Agrobacterium-mediated transformation, a major method for producing stable transformants of *M. polymorpha*, can transform spores [4], thalli [5], and gemmae [6]. This method involves three key steps: (1) pre-culture of *M. polymorpha* tissue; (2) coculture with Agrobacterium harboring recombinant T-DNA; and (3) selection of transformed cells. The AgarTrap method simplifies this process by conducting steps on solid medium in a single Petri dish, applicable to spores (S-AgarTrap), thalli (T-AgarTrap), and gemmae (G-AgarTrap) [7, 8]. Transformation using gemmae and thalli, unlike spores, produces transformants with a uniform genetic background. Among these methods, G-AgarTrap shows the highest transformation efficiency [7, 8].

Antibiotics and herbicides screen transformants by eliminating nontransformed cells. Genes conferring resistance to these agents are used as selection markers. Four common markers in *M. polymorpha* transformation are: *neomycin phosphotransferase II* (*nptII*) for G418/geneticin, kanamycin, and neomycin; *hygromycin B phosphotransferase* (*hpt*) for hygromycin B; *aminoglycoside 3-N-acetyltransferase I* (*aacC1*) for gentamicin; and *mutated acetolactate synthase* (*mALS*) for chlorsulfuron [9]. Hygromycin B, G418, kanamycin, neomycin, and gentamicin are aminoglycoside (AG) antibiotics where the amino groups are conjugated with glycosides. AG antibiotics bind to the bacterial 70S ribosomes, causing mRNA misreading and disrupting protein synthesis [10]. Hygromycin and G418 also bind to 80S ribosomes in eukaryotic cells, while kanamycin, neomycin, and gentamicin, like the majority of AG antibiotics, inhibit the bacterial 70S ribosomes [11, 12, 13]. The herbicide chlorsulfuron inhibits the plant-specific enzyme acetolactate synthase (ALS), which catalyzes the initial step in the biosynthesis of essential branched-chain amino acids valine, leucine, and isoleucine. This inhibition impairs cell division and development, causing plant decay [14], which is overcome by exogenously introduced mALS proteins [14, 15].

In genetic transformation, the effectiveness of selection agents is crucial. Plant sensitivity to antibiotics/herbicides varies by species, tissue/organ, and growth conditions [16]. For example, transformed rice and soybean cells were effectively selected at 50 and 20 μg/ml of hygromycin, respectively [17, 18, 19]. In *Anthoceros agrestis* (hornwort), a relative of *M. polymorpha*, untransformed thallus growth was inhibited by 10 μg/ml hygromycin over three weeks [20]. G418 is commonly used in *M. polymorpha* transformation, while hornwort thallus tissue resists G418 even at 150 μg/ml [20]. For kanamycin, the optimal concentration for selecting transgenic shoot regeneration in apples was 5 μg/ml; concentrations over 10 μg/ml completely inhibited callus growth and shoot primordia formation, even in transformants [21]. Chlorsulfuron inhibited untransformed hornwort thallus growth at 180 ng/ml (0.5 μM) [22], while 100 ng/ml was optimal for selecting transformants in *Camelina sativa* [23].

Determining optimal concentration levels of the selection agent is essential. In our experiments and others, selecting transformants in *M. polymorpha* gemmae transformation has faced issues. Reported concentrations were sometimes ineffective; nontransformed cells continued to grow, or expected transformants either did not grow or died. Determining the minimum concentration to eradicate nontransformed cells and the maximum concentration that resistant plants can tolerate is critical. No comprehensive study on optimal selection agent concentrations for *M. polymorpha* exists. This study investigated the effects of five AG antibiotics (hygromycin, gentamicin, G418, kanamycin, and neomycin) and the herbicide chlorsulfuron at various concentrations on *M. polymorpha* gemmae growth. Additionally, it addresses the cross-activity of resistance enzymes and offers recommendations for selecting agents and their concentrations when introducing multiple selection marker genes into *M. polymorpha*.

## Methods

### Plant materials and growth conditions

This study used the common *M. polymorpha* male accession Takaragaike-1 (Tak-1) as a wild-type [3], which was kindly provided by Dr. Takayuki Kohchi, Kyoto University, Japan and Dr. Shoji Mano, National Institute for Basic Biology, Japan. The Tak1-derived transgenics were resistant to hygromycin, neomycin/kanamycin/G418, gentamicin, and chlorsulfuron harbored plasmids derived from R4pMpGWB139, pMpGWB403, pMpGWB205, and pMpGWB305, respectively [9, 24]. Plants were grown under normal conditions for *M. polymorpha* on half-strength Gamborg B5 salts media (Metck; G5768), 1% sucrose, 2.5 mM MES-KOH (pH 5.7), and 1% Phyto agar (Duchefa Biochemie; P1003), incubated at 22°C under continuous white light at 50 μmol m^−2^ s^−1^.

### Antibiotic/herbicide examinations

For resistance examinations of selection agents, fresh gemmae from 3 to 5 cups of 3- to 4-week-old plants were harvested and pooled in sterile water. Each pooled gemma was then placed individually on 25 mL of solid growth media in a 9 cm diameter petri dish. To evaluate the resistance of an AG resistance gene against other AGs, gemmae were cultured as illustrated in Supplementary Figure S1. Plates were incubated under normal growth conditions for three days. On the third day, antibiotic/herbicide treatments were applied using the G-AgarTrap method [6]. The calculated amount of antibiotic/herbicide for 25 ml of media was prepared in 2 ml of water, mixed thoroughly, and spread evenly over the plate. The solution was absorbed by the medium within a few hours. After 10 more days of growth, images were taken. The selection agents used were hygromycin B (Merck, H3274), kanamycin sulfate (Eproscience, KAN201), G418 disulfate salt (geneticin, Thermo Scientific, J62671), gentamicin sulfate salt (Merck, G1264), neomycin trisulfate salt hydrate (Merck, N6386), and chlorsulfuron (Merck, 34322).

### Protein structural analysis and molecular docking

Molecular graphics and analyses were performed with UCSF ChimeraX [25]. The affinity of AG antibiotics against AAC(3)-Ia protein was determined using AutoDock Vina 1.2.5 provided by SwissDock (swissdock.ch) [26, 27]. The protein model, obtained from the Protein Data Bank (rcsb.org), corresponds to the coenzyme A (CoA)-bound AAC(3)-Ia dimer structure (PDB: 6bvc). All extraneous molecules, except for CoA, were removed from the protein’s 3D structural data using ChimeraX. Molecular information for six AG antibiotics (gentamicin C1, sisomicin, G418, kanamycin A, neomycin B, and hygromycin B) was obtained from PubChem as Simplified Molecular Input Line Entry System (SMILES) codes (pubchem.ncbi.nlm.nih.gov) (Supplementary Table S1). The calculated affinities of twenty configurations for each antibiotic were charted using the R-based boxplot creation tool BoxPlotR (shiny.chemgrid.org).

### Evaluation of aacC1 cross-activity to G418 in tobacco leaves

Agrobacterium lines used to transform *M. polymorpha*, containing plasmids from pMpGWB403 (*nptII* marker) and pMpGWB205 (*aacC1* marker), were cultured overnight. Two milliliters of each culture were centrifuged, and the bacterial pellets were washed and resuspended in 1 mL of sterile water. These suspensions were injected into tobacco leaves using a syringe, followed by a two-day incubation to induce protein expression. Agrobacterium without any vector was injected as a control. Twelve leaf discs (5 mm diameter) were prepared from these leaves and floated on liquid full-strength MS medium, MES-KOH (pH 5.7), containing 100 μg/ml Cefotaxime sodium salt (Fuji Film, 030-16113) to suppress agrobacterial growth, with and without 50 μg/ml G418. The discs were incubated for 7 days under continuous light at 22°C.

### Measurement of chlorophyll contents

Chlorophylls were extracted by immersing each leaf disc (5 mm diameter) in ethanol at room temperature in the dark for 48 hours. Chlorophyll a and b content in the extract was calculated using absorbance values at A664 and A649, according to the Lichtenthaler method [28].

### Imaging and statistical analysis

Total areas were measured using the trainable waikato environment for knowledge analysis (WEKA) segmentation plugin in Fiji/ImageJ (imagej.net/software/fiji). Fresh weights and morphological features were also assessed. Each experiment had at least three biological replicates. Statistical analysis was performed using OriginLab (OriginLab Corporation).

## Results

### Determining the effective concentration of selection agents on M. polymorpha

To evaluate the impact of selection agents on gemmae growth and determine optimal concentrations for *M. polymorpha* transformation, freshly harvested gemmae from wild-type (Tak-1) and lines harboring *nptII* (resistance to neomycin/kanamycin/G418), *aacC1* (gentamicin), *hpt* (hygromycin), and *mALS* (chlorsulfuron) were cultured on half-strength B5 plates with selection agents at various concentrations. After 10 days, total thalli areas, fresh weights, and morphological features were assessed. The efficiency of AG resistance markers (hygromycin, neomycin, kanamycin, G418, and gentamicin) was examined simultaneously (Supplementary Figure S1).

### Hygromycin

**Figure 1:**
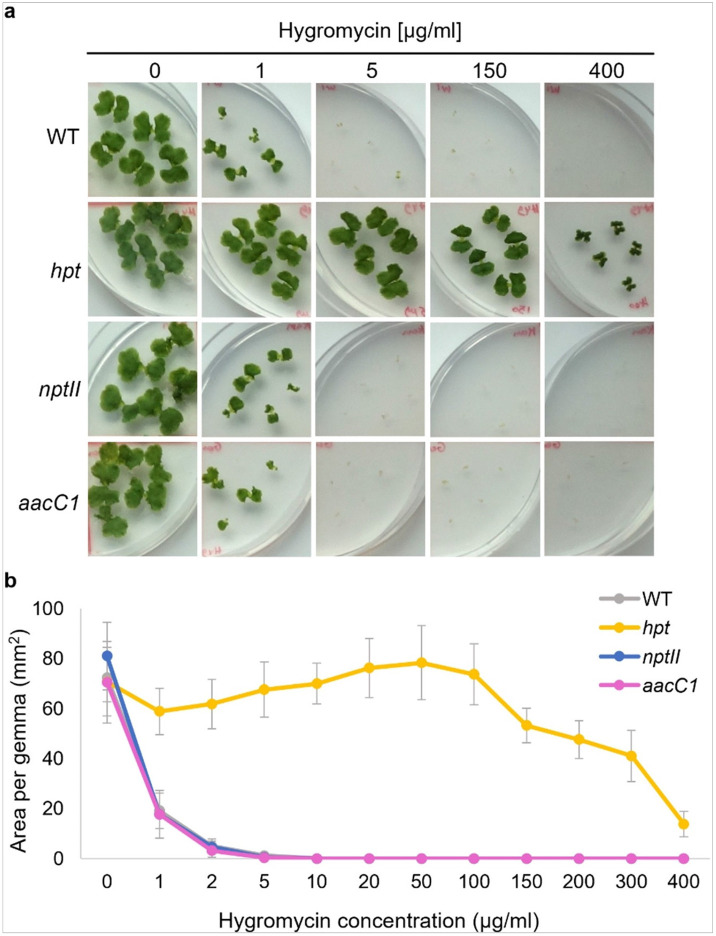
Hygromycin sensitivity of WT and transgenic plants. (a) Morphology of wild type, aacC1 (GenR), nptII (Neo/Kan/G418R), and hpt (HygR) gemmae in various concentrations of hygromycin antibiotic for 10 days. (b) Area of WT and transgenic gemmae in various concentrations of hygromycin. The data represent the mean ± standard deviation of 14–15 gemmae from three biological replicates.

To determine the optimal hygromycin concentration, a range from 1 to 400 μg/ml was added to plates containing 3-day-old gemmae. After 10 days, gemmae were analyzed (Fig. 1, Supplementary Figure S2).

Wild-type gemmae growth was significantly suppressed at 1 μg/ml, and all gemmae were eliminated at 5 μg/ml. Hygromycin-resistant plants (*hpt*) grew well within 1 to 150 μg/ml of hygromycin; however, concentrations above 150 μg/ml significantly inhibited growth and altered morphology (Fig. 1). Thus 5–100 μg/ml is the effective range for hygromycin selection.

### G418

**Figure 2:**
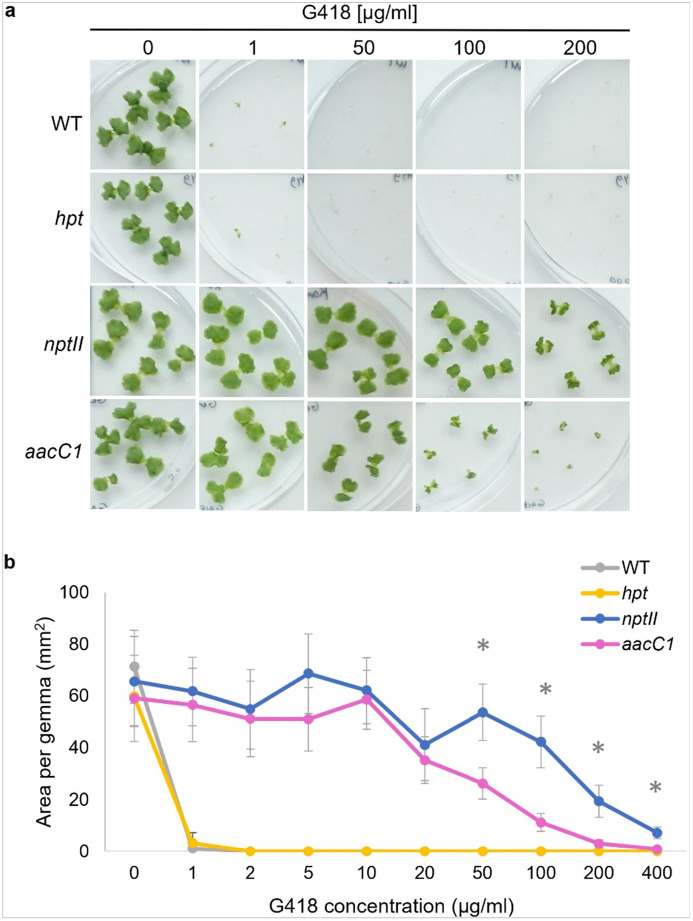
G418 sensitivity of WT and transgenic plants. (a) Morphology of WT, *aacC1* (Gen^R^), *nptII* (Neo/Kan/G418^R^), and *hpt* (Hyg^R^) gemmae in various concentrations of G418 antibiotic for 10 days. (b) Area of WT and transgenic gemmae in various concentrations of G418. The data represent the mean ± standard deviation of 14–15 gemmae from three biological replicates. The significance between *nptII* and *aacC1* is marked with *, indicating p < 0.05, as determined by the Student’s t-test.

G418 concentration was tested from 1 to 400 μg/ml (Fig. 2, Supplementary Figure S3). Even at 1 μg/ml, wild-type and hygromycin-resistant *hpt* gemmae were eliminated. The *nptII* gene, known for G418 resistance, allowed plants to grow normally up to 100 μg/ml and survive at 200 μg/ml, though with morphological changes. Interestingly, *aacC1* plants showed notable G418 resistance, growing normally at 50 μg/ml and barely surviving at 200 μg/ml, albeit smaller than *nptII* plants.

### Kanamycin

**Figure 3:**
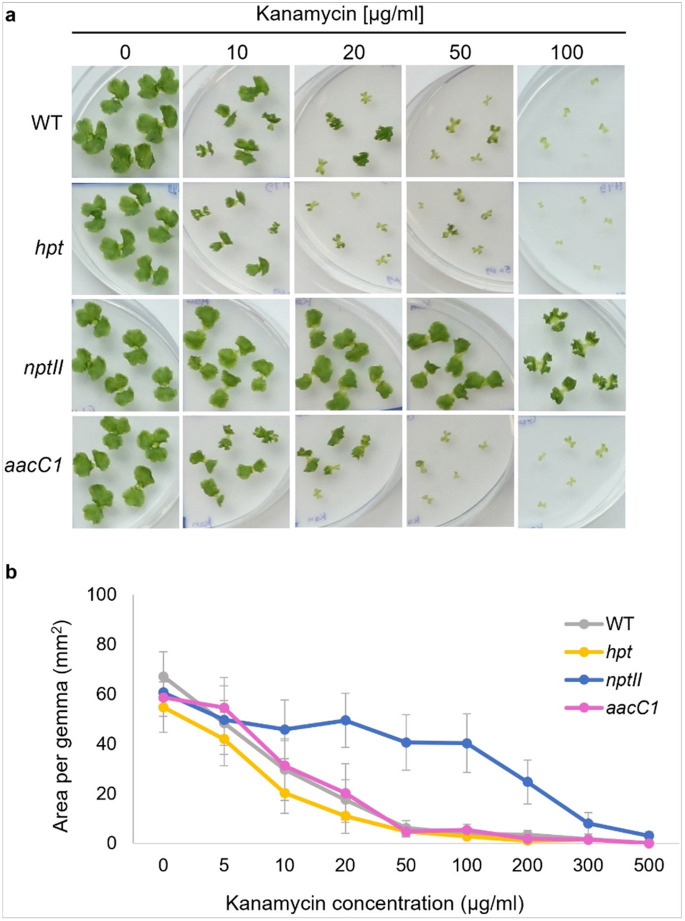
Kanamycin sensitivity of WT and transgenic plants. (a) Morphology of WT, *aacC1* (Gen^R^), *nptII* (Neo/Kan/G418^R^), and *hpt* (Hyg^R^) gemmae in various concentrations of kanamycin antibiotic for 10 days. (b) Area of WT and transgenic gemmae in various concentrations of kanamycin. The data represent the mean ± standard deviation of 14–15 gemmae from three biological replicates.

Kanamycin concentration was tested from 5 to 500 μg/ml (Fig. 3, Supplementary Figure S4). Kanamycin treatment of plants without *nptII* (wild-type, *hpt*, *aacC1*) showed no discernible difference. Growth was affected at 10 μg/ml but remained viable. At 50 μg/ml, nonresistant gemmae survived with severe growth suppression. At 100 μg/ml, all nonresistant gemmae were eliminated, while *nptII* plants survived with shrunken thalli.

### Neomycin

**Figure 4:**
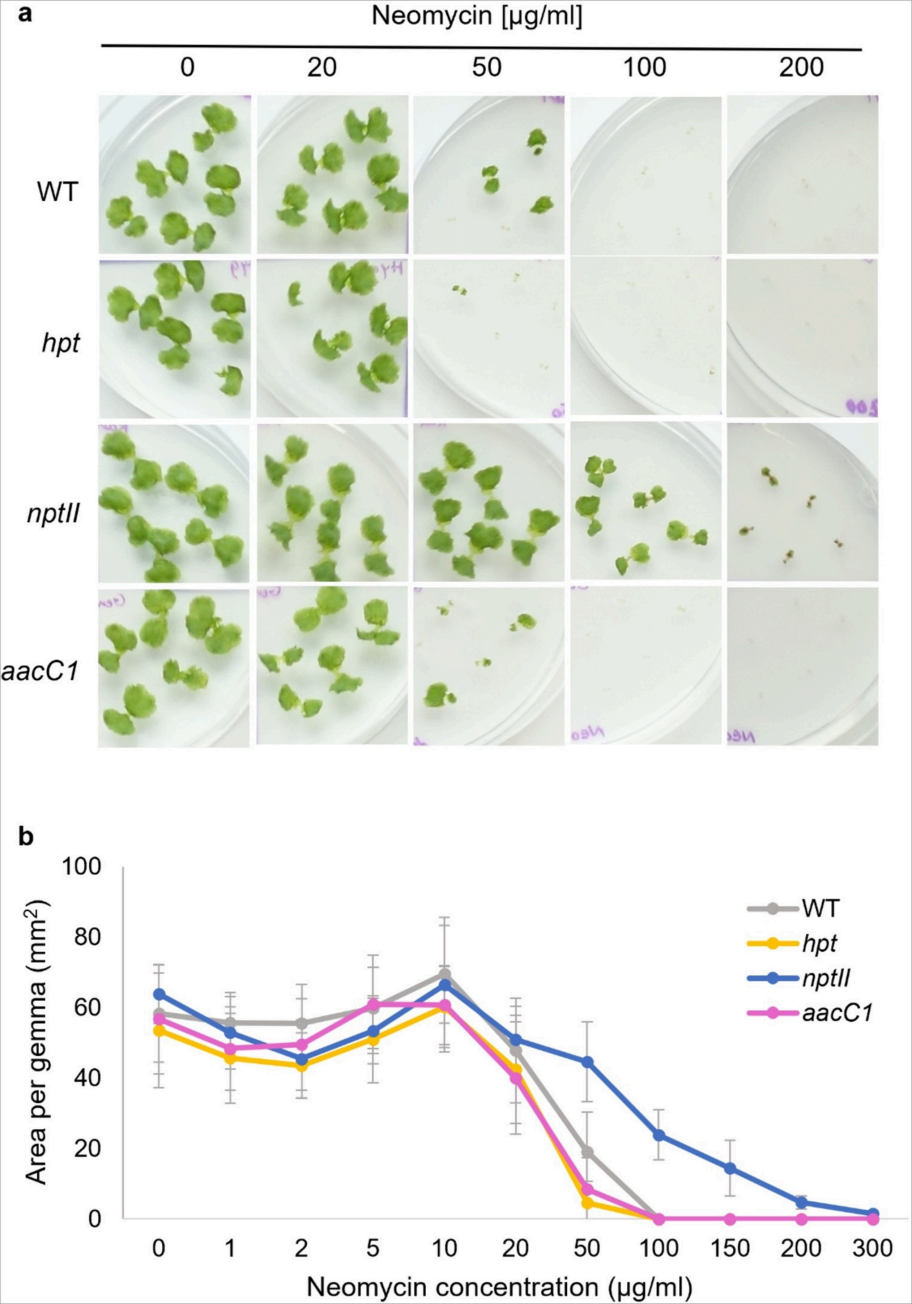
Neomycin sensitivity of WT and transgenic plants. (a) Morphology of WT, *aacC1* (Gen^R^), *nptII* (Neo/Kan/G418^R^), and *hpt* (Hyg^R^) gemmae in various concentrations of neomycin antibiotic for 10 days. (b) Area of WT and transgenic gemmae in various concentrations of neomycin. The data represent the mean ± standard deviation of 14–15 gemmae from three biological replicates.

Neomycin concentration was tested from 1 to 300 μg/ml (Fig. 4, Supplementary Figure S5). Similar to kanamycin, 50 μg/ml was insufficient to eliminate non-resistant plants without *nptII*, which retained their green color. Concentrations above 100 μg/ml successfully eliminated nonresistant plants. Non-*nptII* plants (wild-type, *hpt*, and *aacC1*) showed no difference in response. *nptII* plants survived up to 150 μg/ml but showed severe growth inhibition at higher concentrations.

### Gentamicin

**Figure 5:**
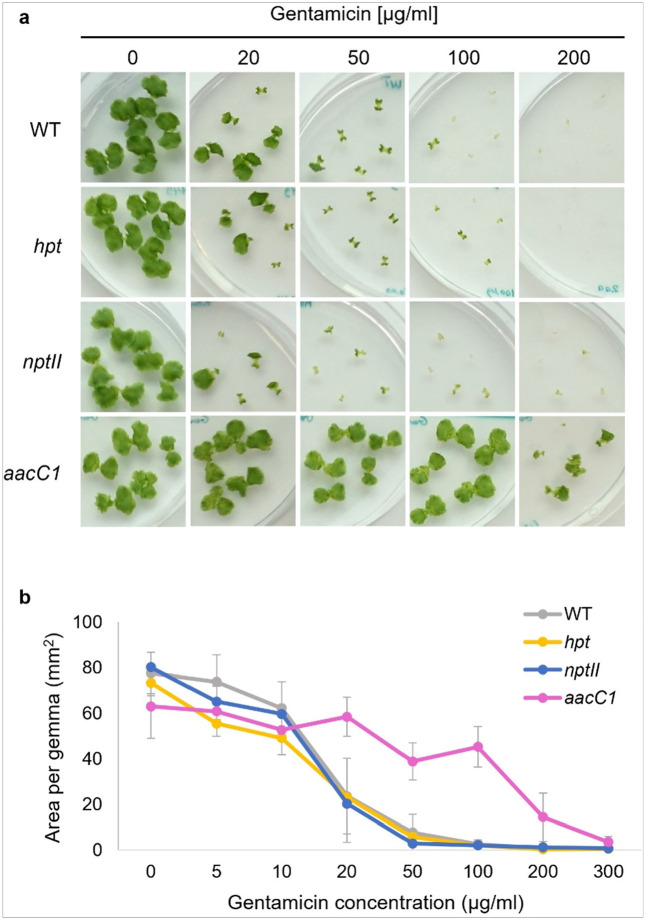
Gentamicin sensitivity of WT and transgenic plants. (a) Morphology of WT, *aacC1* (Gen^R^), *nptII* (Neo/Kan/G418^R^), and *hpt* (Hyg^R^) gemmae in various concentrations of gentamicin antibiotic for 10 days. (b) Area of WT and transgenic gemmae in various concentrations of gentamicin. The data represent the mean ± standard deviation of 14–15 gemmae from three biological replicates.

Gentamicin concentration was tested from 5 to 500 μg/ml (Fig. 5, Supplementary Figure S6). Like kanamycin and neomycin, gentamicin was less effective; nonresistant plants survived at 50 μg/ml but were nearly eliminated at 100 μg/ml. Non-*aacC1* plants (wild-type, *php*, and *nptII*) showed no difference in response. *aacC1* plants grew normally up to 100 μg/ml but were significantly inhibited above 200 μg/ml.

### Chlorsulfuron

**Figure 6:**
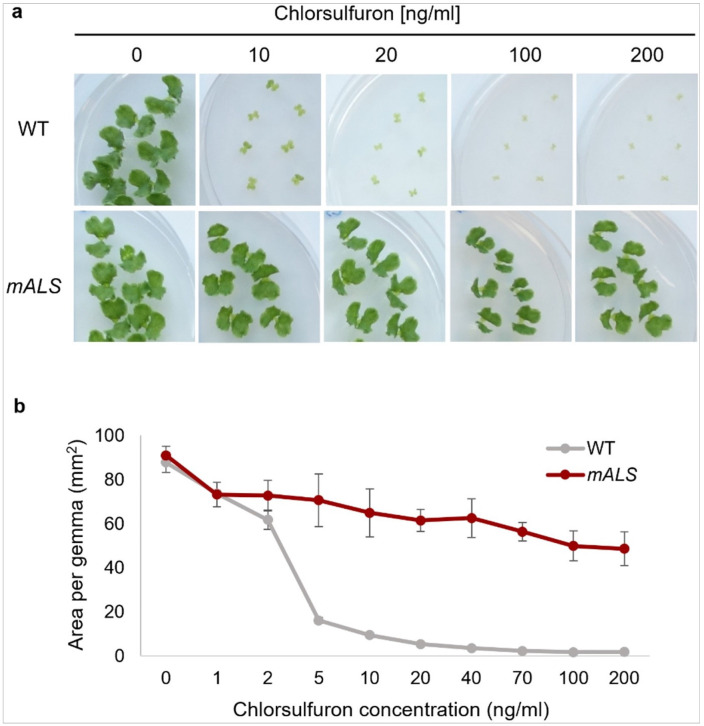
Chlorsulfuron sensitivity of WT and transgenic plants. (a) Morphology of WT and *mALS* (CS^R^) gemmae in various concentrations of chlorsulfuron. (b) Area of WT and transgenic gemmae in various concentrations of chlorsulfuron. The data represent the mean ± standard deviation. n = 3.

Chlorsulfuron concentration was tested from 1 to 1000 ng/ml (0.003 μM to 2.80 μM) (Fig. 6, Supplementary Figure S7). Wild-type gemmae growth was suppressed at 5 to 10 ng/ml, and 20 ng/ml eliminated wild-type plants. Chlorsulfuron-resistant *mALS* plants survived at 200 ng/ml without significant weight reduction and morphological changes. However, at concentrations exceeding 400 ng/ml, the plants survived but exhibited shrunken thalli (Supplementary Figure S7). Thus, chlorsulfuron is effective for *M. polymorpha* gemmae selection at 20–200 ng/ml.

**Figure 7:**
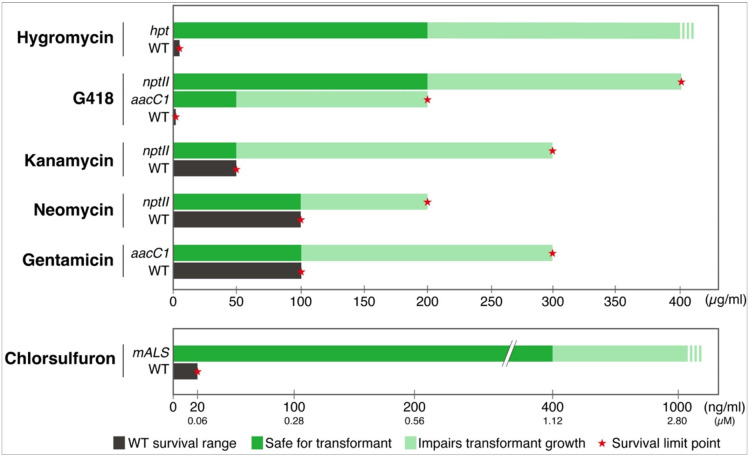
Summary of the effect of selection agents. This bar chart visualizes the results of this study, showing the impact of selection agents on the survival rate of wild type and transgenic lines of *M. polymorpha* gemmae

In summary, these experiments assessed the growth and lethality of *M. polymorpha* gemmae under various AG antibiotic and herbicide concentrations. Comprehensive results are shown in Fig. 7.

### Analysis of predicted bindings between the gentamicin resistance enzyme and AG antibiotics

**Figure 8:**
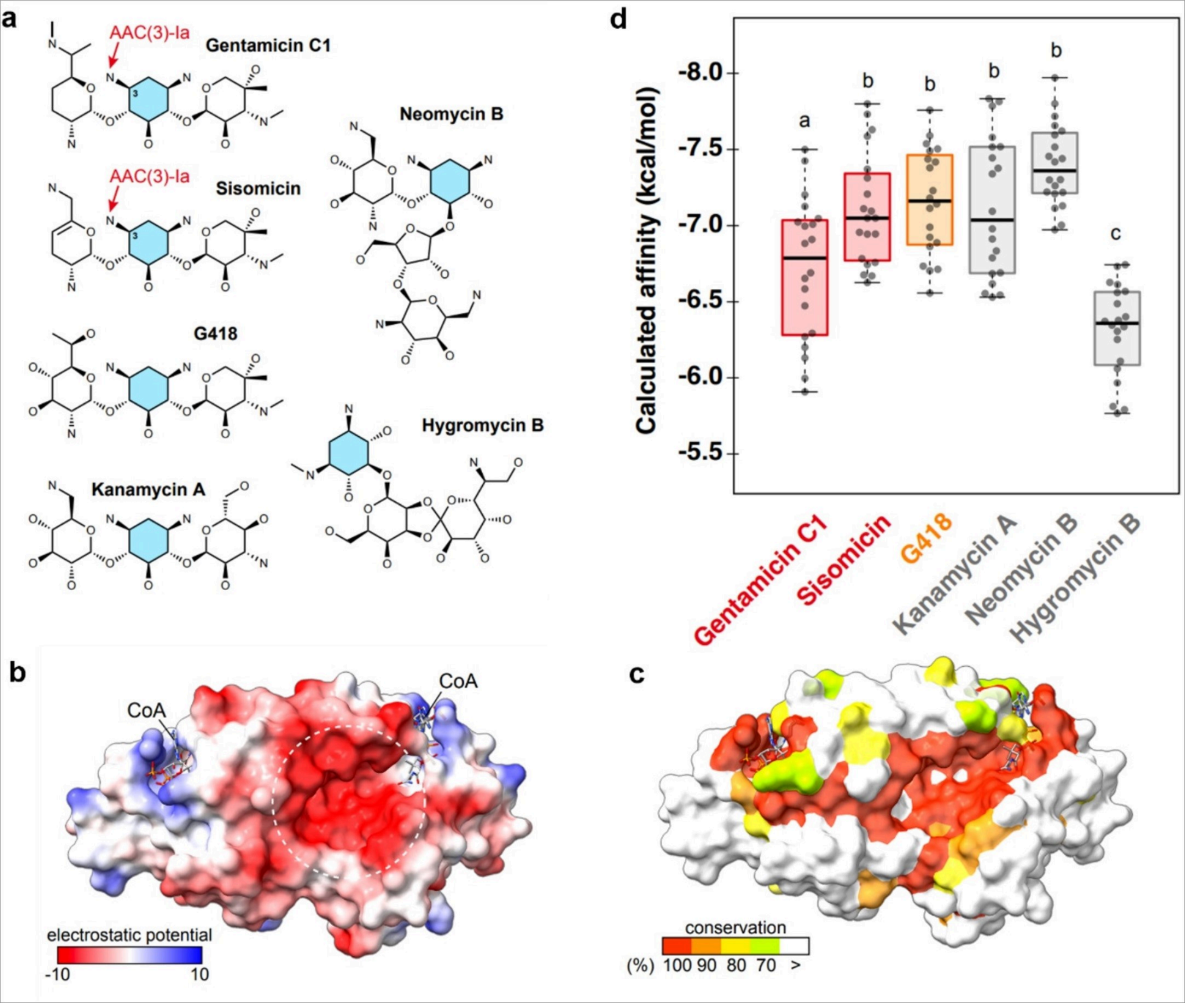
Predicted binding affinity and structural configuration of the gentamicin resistance enzyme with AG compounds. Molecular docking was performed using AutoDock 1.2.5 to evaluate the binding affinity and structural configuration between various AG compounds (Supplementary Table 1) and the 3D structural model of the *aacC1* gene product AAC(3)-Ia; PDB: 6bvc, a dimer bound to coenzyme A (CoA). (a) AG compounds tested by molecule docking. The known amino group where AAC(3)-Ia adds an acetyl group is indicated by a red arrow. The aminocyclitol rings are highlighted in blue. (b) The electrostatic potential of the AAC(3)-Ia protein. The region enclosed by the dashed line corresponds to the negatively charged pocket. (c) The evolutionary conservation of amino acid residues: 100%, 90%, 80%, and 70% in red, orange, yellow, and lime, respectively (Supplementary Figure 8). (d) The calculated binding affinities of the 20 modeled configurations are shown as boxplots with a bee swarm overlay. The known substrate AGs for AAC(3)-Ia (gentamicin and sisomicin) are indicated in red, G418, which showed resistance in this study, is indicated in orange, and the AGs not inactivated by AAC(3)-Ia is indicated in grey. In the boxplot, center lines show medians; box limits represent the 25th and 75th percentiles; whiskers extend 1.5 times the interquartile range, with outliers as dots. Statistical analysis was conducted using one-way ANOVA followed by Bonferroni post-hoc corrections, identifying statistically significant differences at the p < 0.05. n = 20. The actual binding results are shown in Supplementary Figure 9.

During the examination of G418 concentrations, plants with both kanamycin-resistance marker *nptII* and gentamicin-resistance marker *aacC1* showed significant resistance to G418. To understand *aacC1’s* cross-resistance to G418, interactions between AAC(3)-Ia protein (product of *aacC1*) and AG antibiotics were predicted using AutoDock Vina 1.2.5 via SwissDock [29, 26]. Ligands included gentamicin C1, sisomicin, which are known substrates of AAC(3)-Ia, along with G418, kanamycin A, neomycin B, and hygromycin B (Fig. 8a, Supplementary Fig. 9, Supplementary Table 1). The amino group at the 3-position of the aminocyclitol ring in gentamicin and sisomicin, targeted by AAC(3)-Ia, is shared among these AG antibiotics (Fig. 8a) [10]. Molecular docking used the crystal 3D structure of *Serratia marcescens* AAC(3)-Ia protein (Protein Data Bank: 6bvc, dimerized and coenzyme A (CoA)-bound form) [30]. The AAC(3)-Ia amino acid sequence is 99.4% identical between *aacC1* in the pMpGWB205 vector and *S. marcescens*, differing by one amino acid: Val replaced with Leu in *S. marcescens*. The AAC(3)-Ia complex has negatively charged pockets near the CoA binding site (Fig. 8b), conserved among AAC(3)-Ia homologs in the *Pseudomonadota phylum* (Fig. 8c). Molecular docking showed all tested AG antibiotics fit into the negatively charged pocket (Supplementary Figure S9). Neomycin, a non-substrate, exhibited the highest binding affinity, indicating that no correlation between simulated affinity and substrate status (Fig. 8d).

### Evaluation of the aacC1 cross-resistance to G418 in tobacco leaves

**Figure 9:**
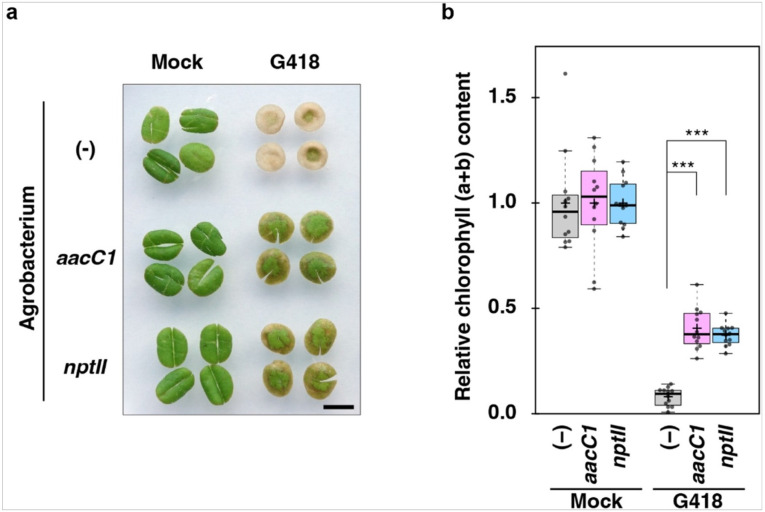
Assay of antibiotic resistance by transient expression of resistance markers in tobacco leaves. (a) Tobacco leaves were injected with Agrobacterium carrying plasmids with either *aacC1* or *nptII* markers, or with Agrobacterium lacking plasmids (−). Two days later, leaf discs were prepared and incubated in a liquid medium with antibiotics 50 μg/ml of G418 for 7 days. Mock indicates without G418. The leaves were notched to make them lie flat for the photograph. Bar = 5 mm. (b) Chlorophyll (a + b) content per leaf disc (Supplementary Table 2) was normalized to the mean value of the treatment with mock, which was set to 1.0. Center line in the box plot represents the median. Whiskers extend 1.5 times the interquartile range from the 25th to the 75th percentiles. Outliers are shown as dots. The cross represents the mean. Statistical significance was assessed using one-way ANOVA followed by Tukey’s HSD with Holm’s correction. Significance of differences between the control (−) and *aacC1* or *nptII:* ***p < 0.001. n = 12.

To determine if *aacC1’s* cross-activity to G418 is specific to bryophytes, we tested this in tobacco *Nicotiana tabacum*. Tobacco leaves were infiltrated with Agrobacterium carrying the same plasmid used for *M. polymorpha* transformants, including either *aacC1* or *nptII* marker. After two days of protein induction, leaves were incubated with G418 for one week. Control leaves died after G418 treatment, while leaves expressing either *aacC1* or *nptII* showed similar resistance to G418. Chlorophyll content was similar in these plants (Fig. 9a, b; Supplementary Table S2).

## Discussion

We assessed the impact of five AG antibiotics (hygromycin, kanamycin, neomycin, G418, and gentamicin) and the herbicide chlorsulfuron on *M. polymorpha* gemmae transformation. Chlorsulfuron, hygromycin, and G418 were the most effective selection markers, eliminating nontransformed plants at low concentrations and offering a broad range of safe concentrations for resistant plants (Fig. 7). While all AG antibiotics bind to bacterial 70S ribosomes and inhibit the protein synthesis process, hygromycin and G418 also inhibit eukaryotic 80S ribosomes. In plants, AG antibiotics like kanamycin and neomycin, which target bacterial ribosomes, can affect the 70S ribosomes within chloroplasts and mitochondria [16, 31]. Gentamicin is generally recognized as a bacterial ribosome inhibitor [11, 12, 13], though recent studies suggest it can bind to eucaryotic ribosomes without causing translation errors [13, 32, 33]. These antibiotics must enter chloroplasts and mitochondria, requiring passage across their double membranes. This passage can be challenging for hydrophilic compounds like AGs [34], potentially facilitated by membrane transport proteins. The chloroplast-localized MAR1 transporter controls the entry of multiple AG antibiotics into chloroplasts in Arabidopsis [31]. The *mar1* mutant shows sensitivity to cytoplasmic-acting antibiotics (including hygromycin and G418) similar to the wild-type but resistance to chloroplast-acting antibiotics (including kanamycin and gentamicin). In our study, G418 and hygromycin exhibited higher lethality compared to kanamycin, neomycin, and gentamicin. G418 and hygromycin effectively eliminated nonresistant plants at low concentrations (1 μg/ml and 5 μg/ml, respectively) without affecting resistant plants even at high concentrations (200 μg/ml). In contrast, kanamycin, neomycin, and gentamicin required higher concentrations to eliminate nonresistant plants (100, 50, and 100 μg/ml, respectively), which also affected resistant plants. Additionally, kanamycin’s dose-response effectiveness is lower in *M. polymorpha* than in other plants, e.g., 25–50 μg/ml is generally used in Arabidopsis transformation [35, 36]. These results suggest that in *M. polymorpha*, these antibiotics have low permeability to organelles or may not easily act on organellar ribosomes. Also, detoxifying proteins from resistance markers may not effectively inactivate these antibiotics due to specific intracellular environments in *M. polymopha*. Although we found that *M. polymorpha* also possesses MAR1 homologs (Mp3g2008, Mp1g18080) by in silico analysis, it is unclear that they contribute to antibiotics translocate. The differences in the effects of these AG groups on *M. polymorpha* are intriguing and warrant further research.

We explored whether AG-resistant genes affect plant responses to other AG antibiotics. Our findings revealed that the gentamicin resistance gene *aacC1* unexpectedly increased plant tolerance to G418 (Fig. 2) but did not affect tolerance to neomycin, kanamycin, or hygromycin (Figs. 1, 3, and 4). Conversely, the *nptII* gene, which confers resistance to G418, kanamycin, and neomycin, did not affect gentamicin tolerance (Fig. 6). AG antibiotic resistance is mediated by AG-modifying enzymes [37]. G418 is known to be inactivated by aminoglycoside 3'-phosphotransferase APH(3’)-II, the product of the *nptII* gene. Surprisingly, the acetyltransferase AAC(3)-Ia, produced by the *aacC1* gene, also conferred G418 resistance in this study. We anticipated an interaction between AAC(3)-Ia and G418, but molecular docking simulations were inconclusive. AG antibiotics, being positively charged, fit into the negatively charged AAC(3)-Ia pocket [38]. The positioning of G418’s functional group within the pocket and its spatial relationship with acetyl-CoA likely play crucial roles in the modification process. However, the exact binding mode between AAC(3)-Ia and AG antibiotics and specific amino acid residues involved remain unknown. While AAC(3)-Ia modifies the 3-position amino group of the aminocyclitol ring in gentamicin and sisomicin, its effect on G418 is uncertain. Detailed enzymatic and structural analyses are needed to fully understand G418 modification by AAC(3)-Ia. AAC(3)-Ia has been reported to confer resistance to gentamicin, sisomicin, and astromicin but not to kanamycin, neomycin, paromomycin, tobramycin, amikacin, or plazomicin [39, 40, 41]. Reports on AAC(3)-Ia’s reactivity to G418 are limited, but some studies are relevant. In the oomycete *Phytophthora palmivora*, the *aacC1* gene conferred resistance to gentamicin but not to G418 [42]. Conversely, in the moss *Physcomitrium patens*, the *aacC1* marker conferred resistance to both gentamicin and G418, but not to kanamycin [43], consistent with our findings in *M. polymopha*. We observed that *aacC1* confers resistance to both gentamicin and G418, but not to kanamycin, neomycin, or hygromycin (Figs. 1-5), although *nptII* confers greater resistance to G418 than *aacC1*. In tobacco, the *aacC1* marker conferred G418 resistance comparable to the *nptII* marker (Fig. 9), suggesting that *aacC1* may confer G418 resistance across various organisms.

## Conclusions

**Figure 10:**
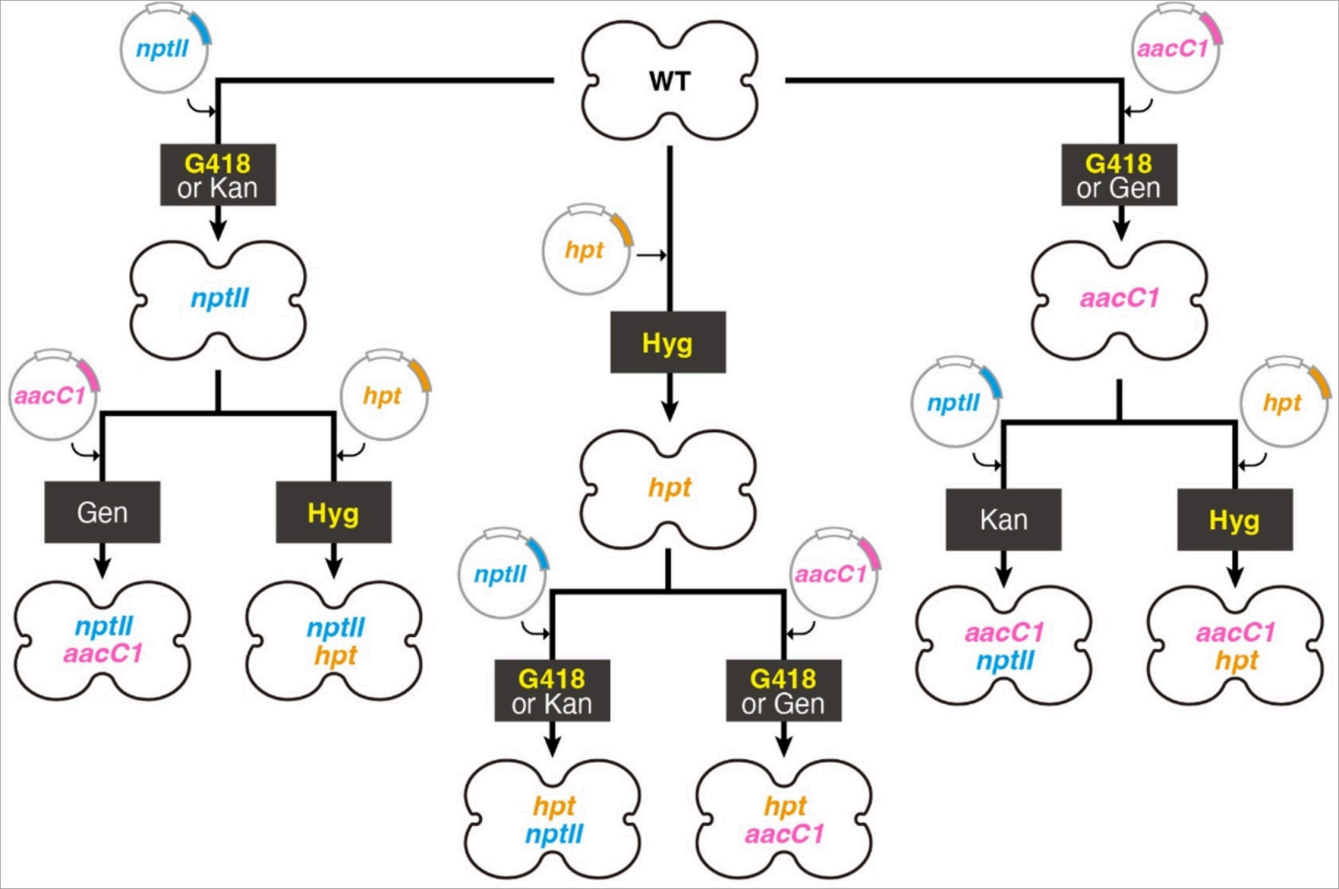
*M. polymorpha* gemmae transformation with AG antibiotics. Antibiotics for the selection of transformed cells, which possess AG resistance markers (*nptII*, *aacC1*, and/or *hpt*), are indicated within black boxes. Antibiotics with a broad selective concentration range are highlighted. Kan: kanamycin, Gen: gentamicin, Hyg: hygromycin.

This study evaluated selection agents for *M. polymorpha* gemmae transformation. Hygromycin, G418, and chlorsulfuron have broad selective concentration ranges, facilitating efficient transformant selection. In contrast, kanamycin, neomycin, and gentamicin require precise concentration settings due to their narrower ranges. For *nptII* marker selection, G418 is preferred over kanamycin or neomycin. While gentamicin is typically used with the *aacC1* marker, G418 can also be effective at 2–50 μ/ml. When introducing multiple constructs (Fig. 10), caution is needed. For instance, if introducing an *nptII* marker into a background with *aacC1*, avoid G418; use kanamycin or neomycin instead, which are not inactivated by the *aacC1* marker. Conversely, if introducing *aacC1* into an *nptII* background, use gentamicin rather than G418. Hygromycin and chlorsulfuron can be combined with any marker without issues. Kanamycin, neomycin, and gentamicin’s narrow ranges can make it challenging to distinguish transformed from nontransformed cells, leading to potential false positives. Therefore, constructs with these agents should include a fluorescent marker for secondary selection. Our study determined optimal selective agent concentrations for *M. polymorpha* gemmae transformation. These recommendations provide valuable insights for enhancing transformation strategies across various organisms.

## Data Availability

All data generated during the selective agent resistance experiments are included in this published article and its supplementary information files. The data generated during the binding simulation of the AAC-Ia enzyme (PDB: 6bvc) with aminoglycoside antibiotics using AutoDock Vina are also included in this article and its supplementary files, with the raw output data available on Zenodo repository, https://doi.org/10.5281/zenodo.14061145.

## Abbreviations

AAC(3)-Ia: aminoglycoside N-acetyltransferase type 3-Ia
aacC1: aminoglycoside 3-N-acetyltransferase I
AG: aminoglycoside
APH(3’)-II: aminoglycoside 3'-phosphotransferase
CS: chlorsulfuron
Gen: gentamicin
hpt: hygromycin phosphotransferase
Hyg: hygromycin
Kan: kanamycin
mALS: mutant Acetolactate Synthase
MAR1: multiple antibiotic resistance1
nptII: neomycin phosphotransferase II
SMILES: simplified molecular input line entry system
WEKA: waikato environment for knowledge analysis
